# Therapeutic Effects of *Bupleurum* Polysaccharides in Streptozotocin Induced Diabetic Mice

**DOI:** 10.1371/journal.pone.0133212

**Published:** 2015-07-15

**Authors:** Lingyu Pan, Hongbo Weng, Hong Li, Zhenzhen Liu, Yanyan Xu, Chunjiao Zhou, Xiaoxiao Lu, Xiaoyu Su, Yunyi Zhang, Daofeng Chen

**Affiliations:** 1 Department of Pharmacology, School of Pharmacy, Fudan University, Shanghai, China; 2 Department of Pharmacognosy, School of Pharmacy, Fudan University, Shanghai, China; University of Catanzaro Magna Graecia, ITALY

## Abstract

Diabetes mellitus is related to low-grade chronic inflammation and oxidative stress. *Bupleurum* Polysaccharides (BPs), isolated from *Bupleurum smithii* var. *parvifolium* has anti-inflammatory and anti-oxidative properties. However, little is known about its therapeutic effects on diabetes. In this experiment, the effects of BPs on alleviation of diabetes and the underlying mechanisms were investigated. Diabetic mice model was established via successive intraperitoneal injections of streptozotocin (100 mg/kg body weight) for two days. Mice with blood glucose levels higher than 16.8mmol/L were selected for experiments. The diabetic mice were orally administered with BPs (30 and 60 mg/kg) once a day for 35 days. BPs not only significantly decreased levels of blood glucose, but also increased those of serum insulin and liver glycogen in diabetic mice compared to model mice. Additionally, BPs adminstration improved the insulin expression and suppressed the apoptosis in pancreas of the diabetic mice. Histopathological observations further demonstrated that BPs protected the pancreas and liver from oxidative and inflammatory damages. These results suggest that BPs protect pancreatic β cells and liver hepatocytes and ameliorate diabetes, which is associated with its anti-oxidative and anti-inflammatory properties.

## Introduction

Diabetes mellitus is manifested with chronic hyperglycemia, impaired lipid and disturbed protein metabolism, due to deficiency of insulin secretion and/or insulin resistance in peripheral tissues [1]. Diabetes is associated with a chronic low-grade inflammation [2, 3], which plays a key role not only in the progress of diabetes, but also in its late vascular complications. Thus, anti-inflammatory properties of traditional glucose-lowering drugs may contribute to antidiabetic effects [3–5]. Diabetes mellitus is also associated with increased oxidative stress [6, 7]. Oxidative stress causes cell dysfunction through multiple pathways, especially β-cells in pancreas [8].

Many Chinese herbal medicine formulas have been proved to have anti-inflammatory and anti-oxidative characteristics, as well as glucose-lowering effects in diabetic patients [9–12]. Among them, some polysaccharide-containing herbs can protect the functions of islet β-cells and subsequent insulin production [13, 14].

Radix Bupleuri, known as ‘Chai-Hu’, is dried roots of *Bupleurum chinense* or *Bupleurum scorzonerifolium* [15]. Radix Bupleuri is frequently used to treat inflammatory diseases in the prescriptions of traditional Chinese medicine [16, 17]. All these studies suggested that ‘Chai-Hu’ could have potential benefits in treating diabetes and diabetic complications. *Bupleurum* Polysaccharides (BPs), extracted from *Bupleurum smithii var*. *parvifolium*, has been demonstrated to possess pharmacological activities, including anti-inflammation [18], anti-oxidation [19], kidney protection [20, 21] and Toll-like receptor 4 modulation [22]. The present study is to investigate the antidiabetic properties and the mechanisms of BPs in STZ-induced diabetic mice.

## Materials and Methods

### 1.1 Isolation and characterization of *Bupleurum* polysaccharides

**Fig 1:**
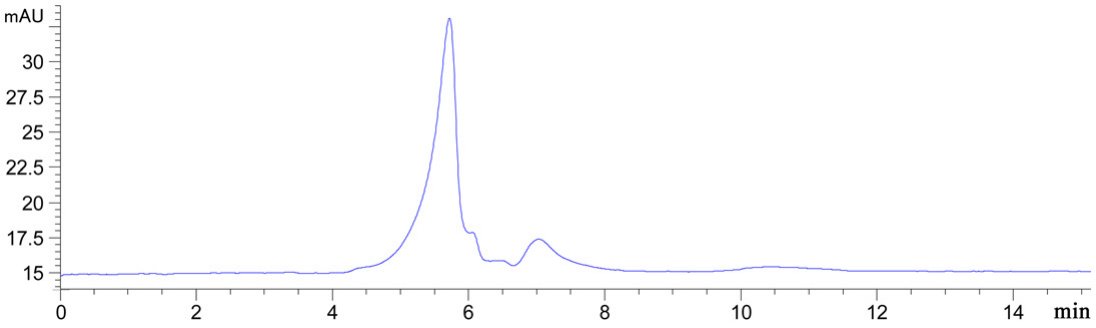
HPGPC chromatogram of the *Bupleurum polysaccharides* gained with a TSK GMPWXL gel filtration column (7.6 mm × 300 mm, TOSOH), eluted with ultrapure water at 1.0 mL/min, and detected at 254 nm.

The roots of *Bupleurum smithii var*. *parvifolium*, which is categorized as DFC-CH-H2003121602 in the Herbarium of Materia Medica, Department of Pharmacognosy, School of Pharmacy, Fudan University, Shanghai, People’s Republic of China, were purchased from Shanghai Hua-Yu Chinese Materia Medica Co. Ltd. Crude polysaccharides were extracted and characterized as previously described [15, 18]. The crude polysaccharides contained one major polysaccharide with few minor ones, which were characterized by high performance gel permeation chromatography (HPGPC) (Fig 1)

### 1.2 Animals and ethics statements

Eight-week-old male C57BL/6 mice were purchased from Slaccas-Shanghai Lab Animal Ltd. (SPF II Certificate; No.SCXK2012-0002), and kept under specific pathogen-free and normal housing conditions in a 12-h light and dark cycle. All mice received humane care in compliance with the Guide for the Care and Use of Laboratory Animals of the National Institutes of Health. All the protocols in this experiment were approved by the Animal Ethical Committee of School of Pharmacy, Fudan University (Permit Number: 2013–1). All operations were performed under ethyl carbamate anesthesia, and all efforts were taken to minimize suffering.

### 1.3 Induction of diabetes in mice

After one week of being housed in a new environment, the C57BL/6 mice were fasted but free for water for 16 h before the induction of diabetes. Streptozotocin (STZ) (Sigma, MN, USA), freshly prepared in buffer solution (0.1mol/L sodium citrate and 0.1 mol/L citric acid, pH 4.2–4.5), was intraperitoneally injected into the mice in a dose of 100 mg/kg for two successive days (day1 and day2). The diabetes induction method was mildly modified according to the reference [23]. After 72 h, the mice display polydipsia and polyuria. After one week (day 9), postprandial blood glucose levels were measured by blood glucose test strips (Abbott). Mice with blood glucose levels higher than 16.8mmol/L were defined diabetic and chosen for experiments. Ten sex- and age-matched mice were intraperitoneally injected with the buffer solution as the controls.

### 1.4 Treatment

Diabetic mice were allocated into 4 groups randomly (n = 8–12 per groups): vehicle-treated diabetic mice (model group); BPs (30mg/kg and 60mg/kg) treated diabetic mice; glyburide (7.2mg/kg) treated diabetic mice. BPs was ground, suspended in normal saline, and orally administered to the diabetic mice once daily for 35 days. Glyburide (Weibian, Shanghai, China) was suspended in 0.5% sodium carboxymethyl cellulose (CMC) for administration as a positive control. The normal mice in control group received normal saline orally. All the mice were on common pellet diet (Shanghai Shilin, cat Q/TJCX-2010) during the study. The ingredients of the pellet met China national standards, such as GB-T6435-2006.

Body weight was measured every two days during the study. Postprandial urine was collected for 5 hours via metabolism cage in which mice could freely access to water at day 36 and 37 (BPs treatment for 27 and 28 days). At the endpoint of the treatments, postprandial blood was harvested via orbital sinus and centrifuged at 800g for 15min to collect serum. Serum, liver and pancreas were collected for molecular or histological assessments.

### 1.5 Biochemical assays in serum and urine sample

Serum glucose, total cholesterol (TC), triglyceride (TG), high density lipoprotein cholesterol (HDL-C), low density lipoprotein cholesterol (LDL-C), and urine glucose were measured using assay kits, following manufacturers’ instructions (Fenghui, Shanghai, China). Serum insulin concentrations were tested using an enzyme-linked immunosorbent assay (Millipore mouse insulin kit).

### 1.6 Histopathological examination

Pancreas and liver were fixed in 10% formaldehyde and embedded in paraffin. The specimens were processed to obtain 4-μm-thick paraffin sections. The tissue sections were stained with hematoxylin and eosin (HE). Liver sections were also stained by periodic acid-Schiff’s (PAS) method.

### 1.7 Immunohistochemistry

The streptavidin-biotin-peroxidase complex (SABC) method was utilized to determine the insulin expression in the pancreas [24, 25]. The sections were deparaffinized, rehydrated and blocked of endogenous peroxidase activity. After being blocking with 5% BSA, the sections were incubated with rabbit monoclonal anti-insulin antibody (1:200, BS1511, Bioworld) overnight. The sections were treated with biotinylated anti-rabbit immunoglobulin G (Maixin, China) and SABC reagent incubation. Staining was visualized by incubation with 3,3′-diaminobenzidine-tetrahydrochloride (Changdao, China). The sections were counterstained with hematoxylin and examined under a light microscopy (Leica Inc. Switzerland).

### 1.8 Apoptotic assay

Tissue sections were deparaffinized and rehydrated. After processed with proteinase K (20 μg/ml) for 15 min at 37°C, the sections were treated with the terminal deoxynucleotidyl transferase-mediated dUTP nick-end labeling (TUNEL) reagents (Beyotime, Jiangsu, China) at 37°C for one hour, counterstained with DAPI (4',6-diamidino-2-phenylindole, Pierce Chemical Co.), and analyzed under a fluorescent microscope (Leica Inc. Switzerland). The cells with green fluorescence were defined as apoptotic cells.

### 1.9 Western blot assay

Pancreas protein was homogenized in lysis reagent (Beyotime, Shanghai, China) and incubated on ice for 30 min. After centrifugation at 10,000g for 15 min at 4°C. The supernatants were collected and total protein was measured using the bicinchoninic acid assay (Beyotime, Shanghai, China). Samples were separated by SDS-PAGE and transferred to PVDF membranes (Millipore, MA, USA). Membranes were blocked with 5% skim milk for 1h. Blots were probed with primary antibodies for caspase 3, caspase 8 (Abcam, Britain), and GAPDH (Bioworld, China) overnight at 4°C. Then, membranes were incubated with peroxidase-labeled secondary antibodies for 3 h at room temperature. All signals were visualized by enhanced electrochemiluminescence (ECL) reagent (Beyotime, Shanghai, China) and captured with a camera-based imaging system (FluorChem SP, CA, USA).

### 1.10 Determination of liver glycogen by anthrone-sulfuric acid assay

Liver glycogen content was determined by anthrone-sulfuric acid assay kit (Nanjing Jian Cheng, Nanjing, China). Liver tissues were hydrolyzed by alkaline liquor for 20 min at 100°C [tissue weight (g): alkaline liquor (mL) = 1:3]. Glycogen hydrolysate was diluted with deionized water and mixed with color reagent for 5min at 100°C. The absorbance of the mixtures was measured at a wave length of 620nm. The glycogen content was calculated based on a standard curve.

### 1.11 Determination of oxidative stress and inflammatory markers in liver and pancreas tissues

Livers tissues were homogenized in normal saline and centrifuged at 10000g for 15 min at 4°C. The supernatants were immediately used for assays of superoxide dismutase (SOD), malondialdehyde (MDA) and catalase (CAT) using assays kits, following manufacturers’ instructions (Nanjing Jian Cheng, Nanjing, China). Pancreas tissues were homogenized in lysis buffer and centrifuged at 10000g for 15 min at 4°C. The supernatants were assayed for SOD and CAT.

TNF-α and IL-6 in liver and pancreas supernatants were measured using the ELISA kits (Boatman, Shanghai, China). Protein concentration was determined by Coomassie brilliant blue test, with bovine serum albumin as the standard [26].

### 1.12 Statistical analysis

Quantitative variables were expressed as means ± S.D. One-way analysis of variance (ANOVA) was used to analyze the differences between groups. If any significant changes were found, post hoc comparisons were performed using Fisher’s PLSD. *P*-value <0.05 was considered significant.

## Results

### 2.1 Effect of BPs on body weight in STZ-induced diabetic mice

**Fig 2:**
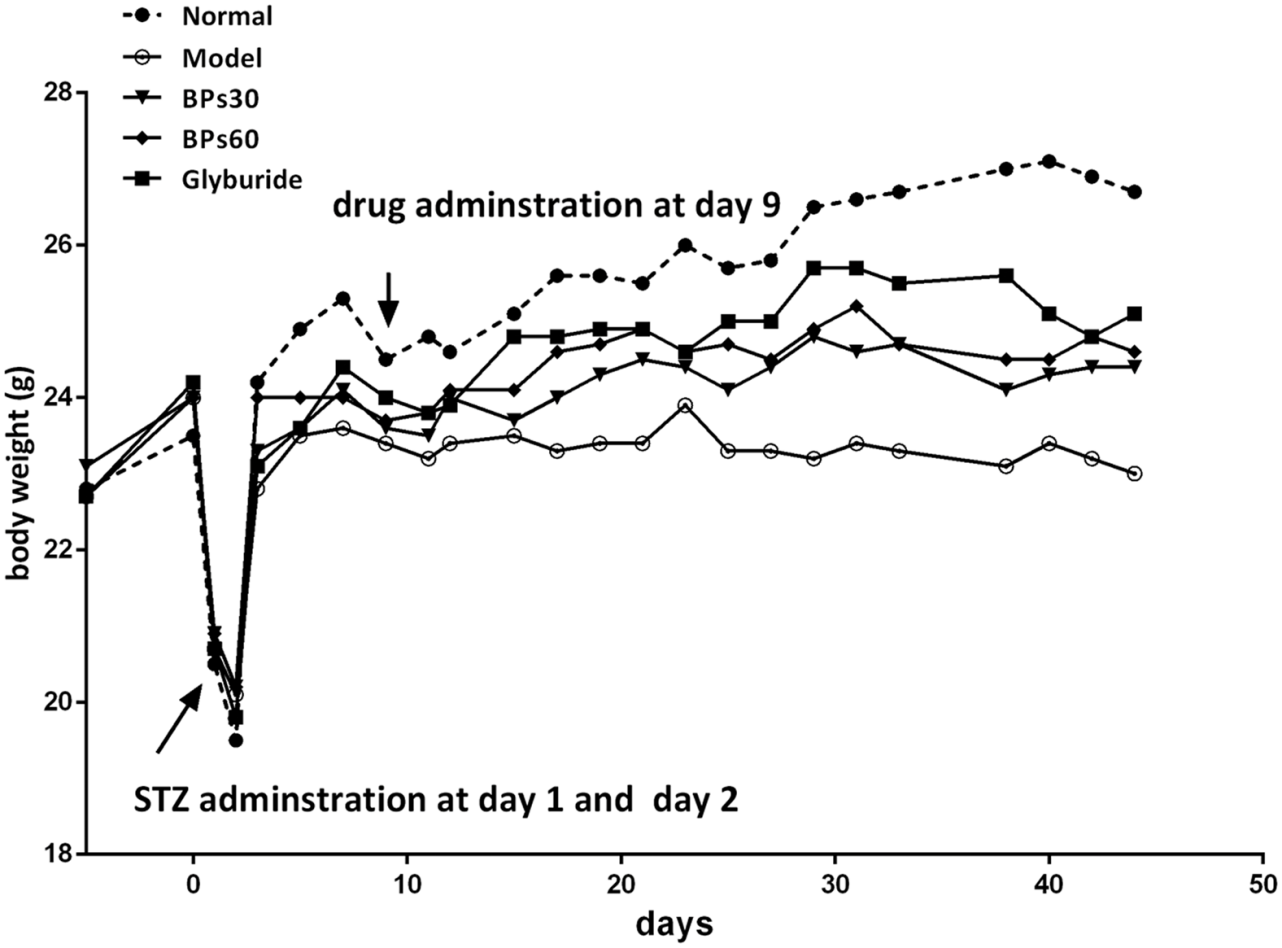
Effect of BPs on body weight of STZ-induced diabetic mice. Diabetic mice were treated with BPs (30 and 60 mg/kg/day), glyburide (7.2 mg/kg/day), or vehicle from day 9 to day44. Data were expressed as means ± S.D.; *P<0.05, **P<0.01, ***P<0.001 were compared with vehicle-treated group, analyzed by one way ANOVA and the Fisher’s PLSD.

Body weights of the mice in all groups were similar at the beginning of the experiments (Fig 2). Successive injections of STZ on day 1 and 2 caused rapid and significant decreases in body weight. While the mice recovered from the initial STZ treatment, the regain of body weight in the diabetic mice was significantly lower than that in the normal mice from day 11 to day 44 (*P*<0.001). After treatments of BPs and glyburide from day 27 to day 35, the regain of body weight was significantly greater in the BPs- and glyburide-treated group than that in the vehicle-treated diabetic group (*P*<0.05).

### 2.2 Effect of BPs on glucose metabolism

**Fig 3:**
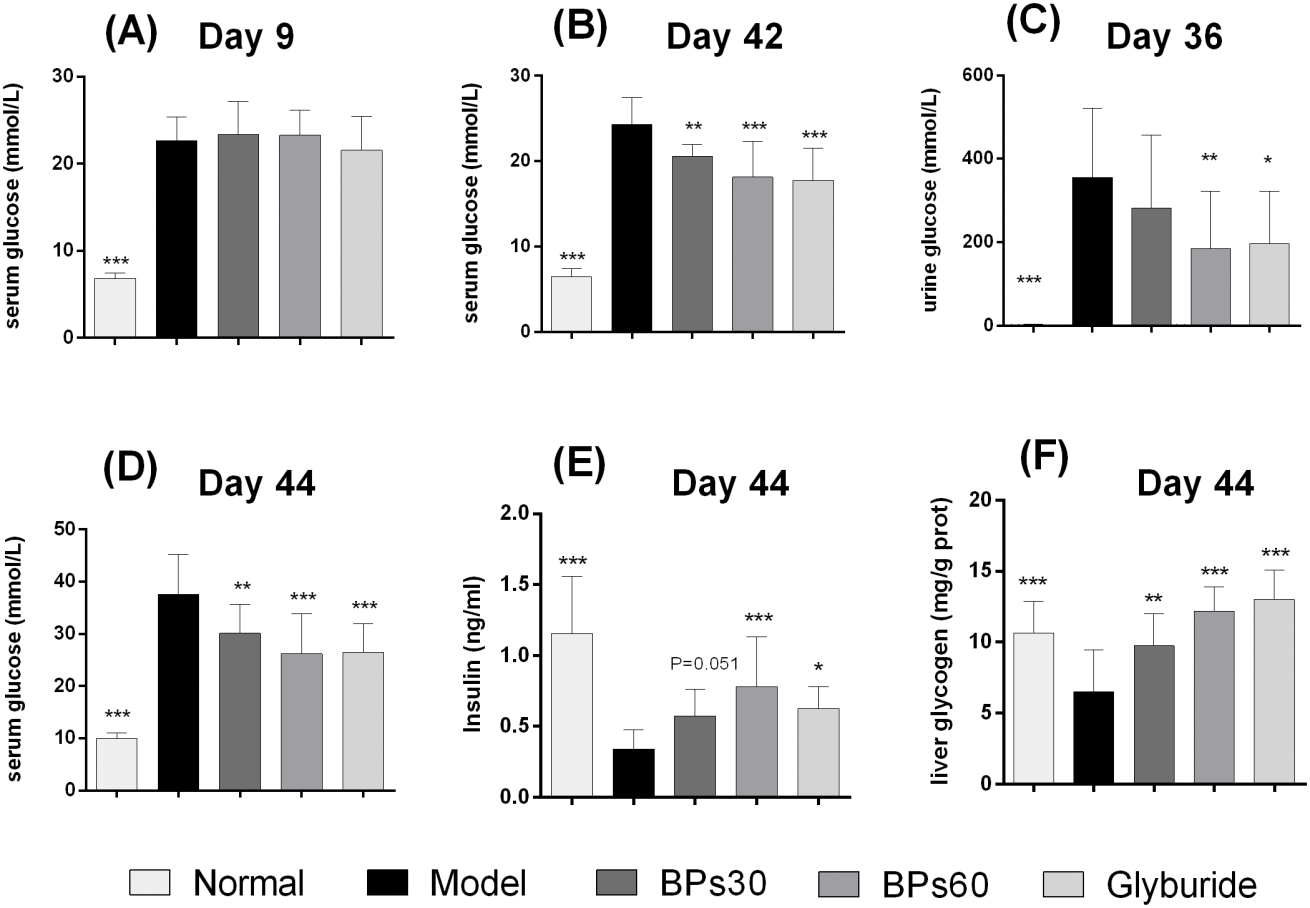
Effect of BPs on glucose metabolism. Diabetic mice were treated with BPs (30 and 60 mg/kg/day), glyburide (7.2 mg/kg/day), or vehicle for 35 days. The levels of serum glucose (A, B) were determined by blood glucose test strips, while the levels of urine glucose (C) and serum glucose (D) were tested by biochemical methods. Data were expressed as means ± S.D.; *P<0.05, **P<0.01, ***P<0.001 were compared with model group, analyzed by one way ANOVA and the Fisher’s PLSD.

During the experiment, some pilot experiments were done to determine the effects of BPs on glucose level. At day 9, the postprandial blood glucose levels of the STZ-induced mice was significantly greater than those of normal mice (>16.8 mmol/L, *P*<0.001), defined that diabetes had been established (Fig 3A). The beneficial effects of BPs were observed on lowering postprandial urine glucose at day 36 (Fig 3C) and serum glucose at day 42 (Fig 3B) separately.

At the end point (day 44), the diabetic mice exhibited significant increase in the levels of postprandial blood glucose (*P*<0.001), compared with those in the normal mice. Administration of BPs or glyburide significantly decreased the glucose levels (*P*<0.01), compared with those in the vehicle-treated group (Fig 3D).

Compared to the control group, the model mice exhibited significant decrease in the levels of serum insulin (*P*<0.001). However, treatments with BPs at 60 mg/kg and glyburide at 7.2 mg/kg significantly increased the levels of serum insulin (*P*<0.05), compared with those in the model mice (Fig 3E).

The levels of liver glycogen in diabetic mice were significantly lower than those in the normal mice (*P*<0.001). BPs and glyburide significantly increased glycogen content in diabetic mice (*P*<0.01), compared with vehicle-treated diabetic mice (Fig 3F).

### 2.3 Effect of BPs on the blood lipid profile

The levels of LDL-C (*P*<0.001), TC (*P*<0.01), and TG (*P*<0.001) were significantly increased; whereas, the levels of serum HDL-C (*P*<0.05) were significantly decreased in the diabetic mice, compared with the normal mice (Table 1). BPs and glyburide treatments significantly decreased the LDL-C levels (*P*<0.05), compared with the vehicle-treated group. BPs at 30 mg/kg and glyburide significantly increased the levels of HDL-C (*P*<0.05), compared with those in the vehicle-treated group. At the levels of TC and TG, glyburide only significantly decreased TC (*P*<0.05), while BPs exerted no significant effect on the TC and TG.

**Table 1:** Effect of BPs on serum LDL-C, HDL-C, TC and TG of diabetic mice.

Group	LDL-C(mmol/L)	HDL-C(mmol/L)	TC(mmol/L)	TG(mmol/L)
Normal	2.18 ± 0.15***	3.68±0.26*	3.38±0.35**	1.01±0.27***
Model	2.70 ± 0.32	3.30±0.47	4.19±0.84	2.78±1.32
BPs30	2.48 ± 0.14*	3.66±0.20*	3.85±0.19	1.97±0.74
BPs60	2.39 ± 0.18**	3.59±0.32	3.86±0.35	2.27±1.10
Glyburide	2.40±0.18**	3.65±0.42*	3.69±0.56*	2.02±0.83

NOTE: Diabetic mice were treated with BPs (30 and 60 mg/kg/day), glyburide (7.2 mg/kg/day), or vehicle for 35 days. Data were expressed as means ± S.D.;

*P<0.05,

**P<0.01,

***P<0.001 were compared with model group, analyzed by one way ANOVA and the Fisher’s PLSD.

### 2.4 Effect of BPs on pathology and insulin expression in pancreas of diabetic mice

**Fig 4:**
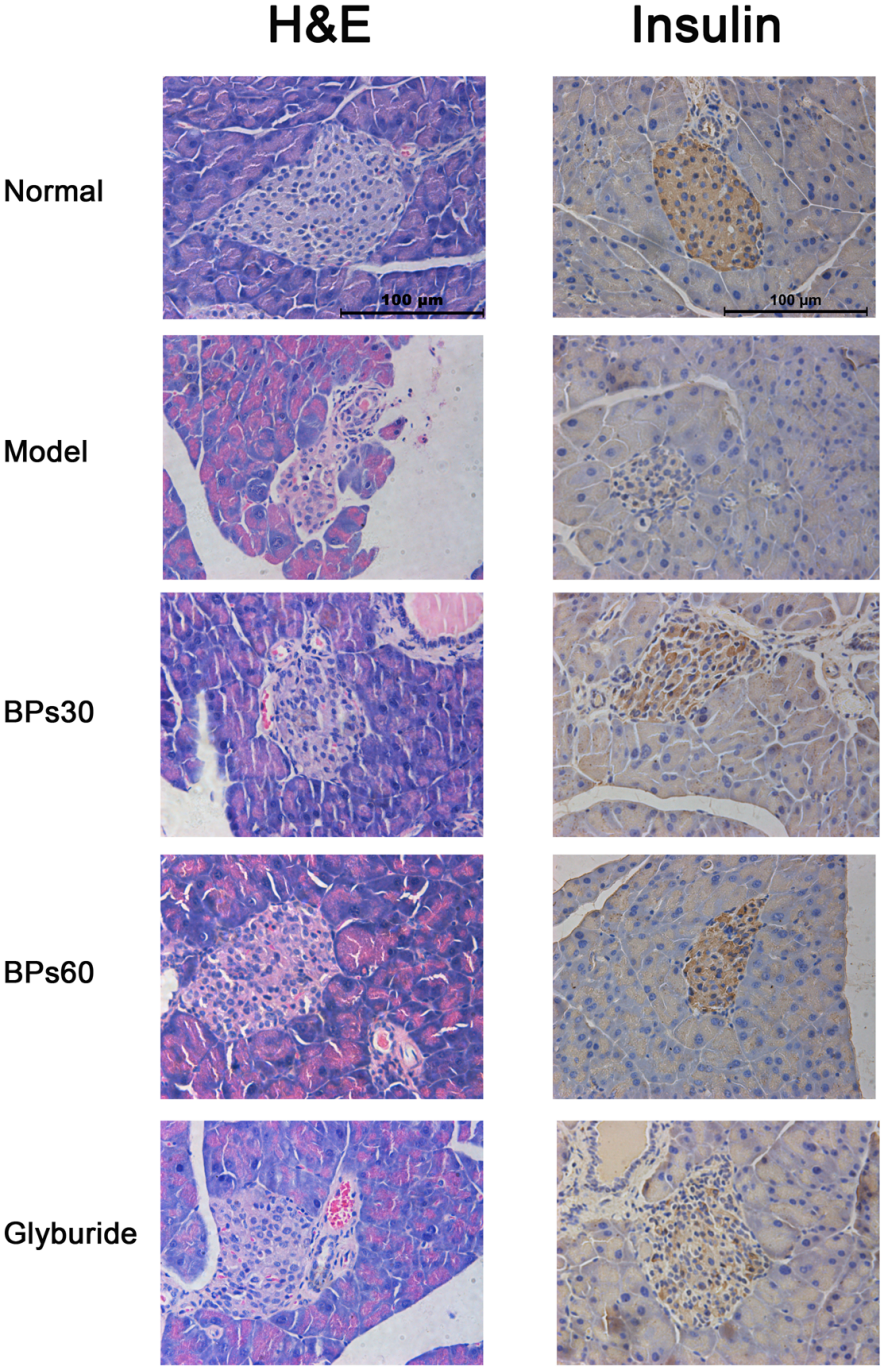
Effect of BPs on pathology and insulin expression in pancreas of diabetic mice. Light microscopy 400×. Diabetic mice were treated with BPs (30 and 60 mg/kg/day), glyburide (7.2 mg/kg/day), or vehicle for 35 days.

At the histological level, the Langerhans’ islets of the pancreas exhibited normal circular morphology with healthy cell lining in the normal mice. The exocrine acini portion of the islets was well organized and normal morphology. The islet cells also appeared in normal morphology (Fig 4). In contrast, in the vehicle-treated diabetic mice, the pancreas tissues showed shrunken islets and the islet cell mass appeared condensed and disorganized. However, in the groups treated with BPs or glyburide, the Langerhans’ islets were larger than those in the vehicle-treated diabetic mice.

Immunohistochemistry showed conclusive insulin positivity in the pancreatic islets of the normal mice (Fig 4). The exocrine pancreas were completely negative for insulin. Few scattered cells were immunopositive for insulin in the vehicle-treated diabetic mice. However, diabetic mice treated with the BPs and glyburide showed more rich insulin-positive staining compared with the vehicle-treated diabetic mice, although the positively stained area were still fewer than those of the normal mice.

### 2.5 Effect of BPs on cell apoptosis in pancreas of diabetic mice

**Fig 5:**
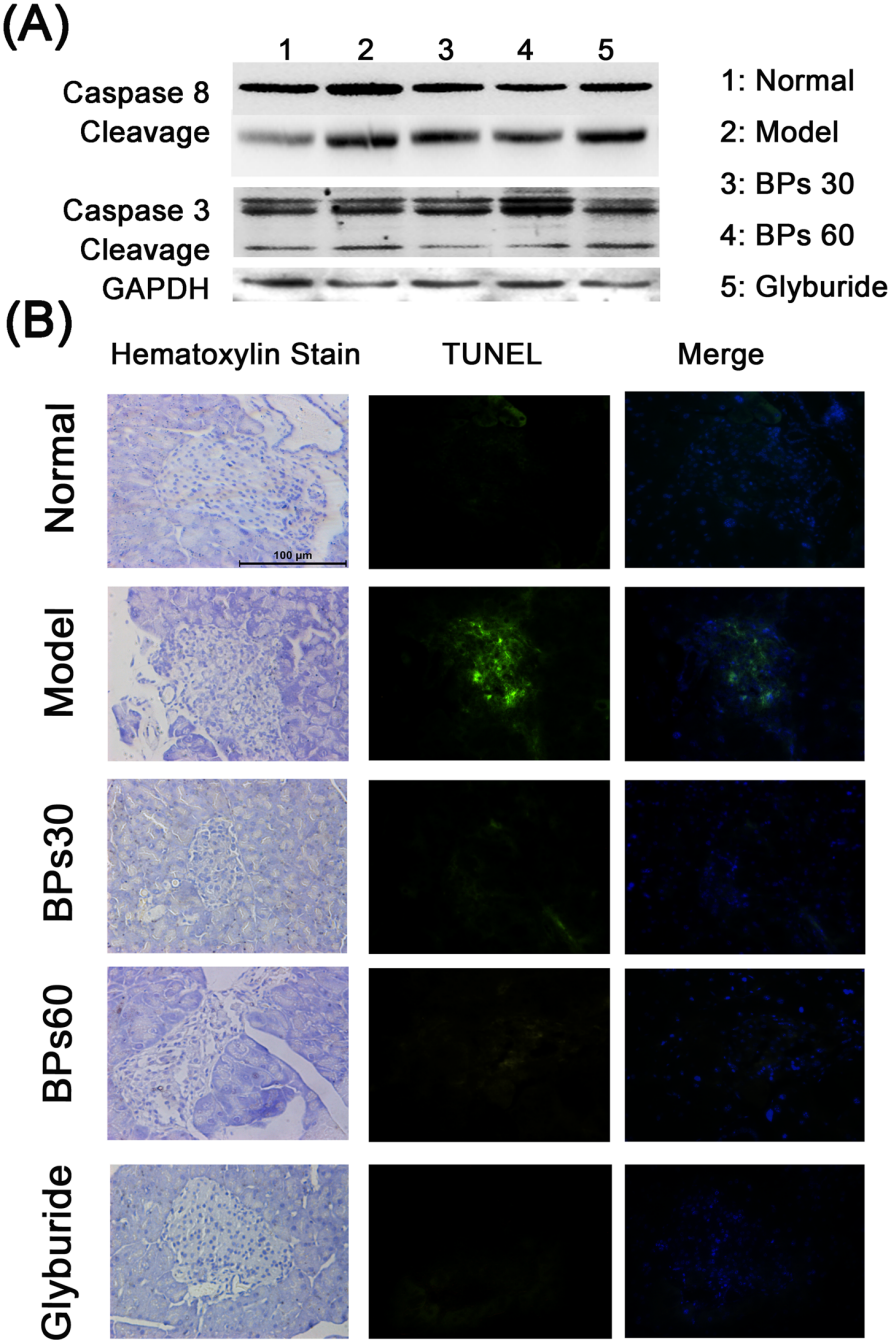
Effect of BPs on cell apoptosis in pancreas of diabetic mice. Diabetic mice were treated with BPs (30 and 60 mg/kg/day), glyburide (7.2 mg/kg/day), or vehicle for 35 days. Panel (A) shows western blot analysis of protein expression. Panel (B) shows the TUNEL analysis in different groups. Blue fluorescence represents the cell nucleus and green fluorescence indicates TUNEL-positive cells. Florence and Light microscopy 400×. These sections of each group were from successive sections.

As shown in the Fig 5A, the caspase 8 and caspase 3 cleavage forms elevated markedly in pancreas of STZ induced diabetic mice, compared to the normal mice. However, BPs treatment suppressed the expression of the cleavage forms in pancreas.

Markedly increased TUNEL signals (apoptosis) were observed in the vehicle-treated diabetic mice, compared with those in the normal mice. Treatments with BPs or glyburide significantly reduced the levels of apoptosis in the pancreas islets (Fig 5B).

### 2.6 Effect of BPs on histopathology of liver in diabetic mice

**Fig 6:**
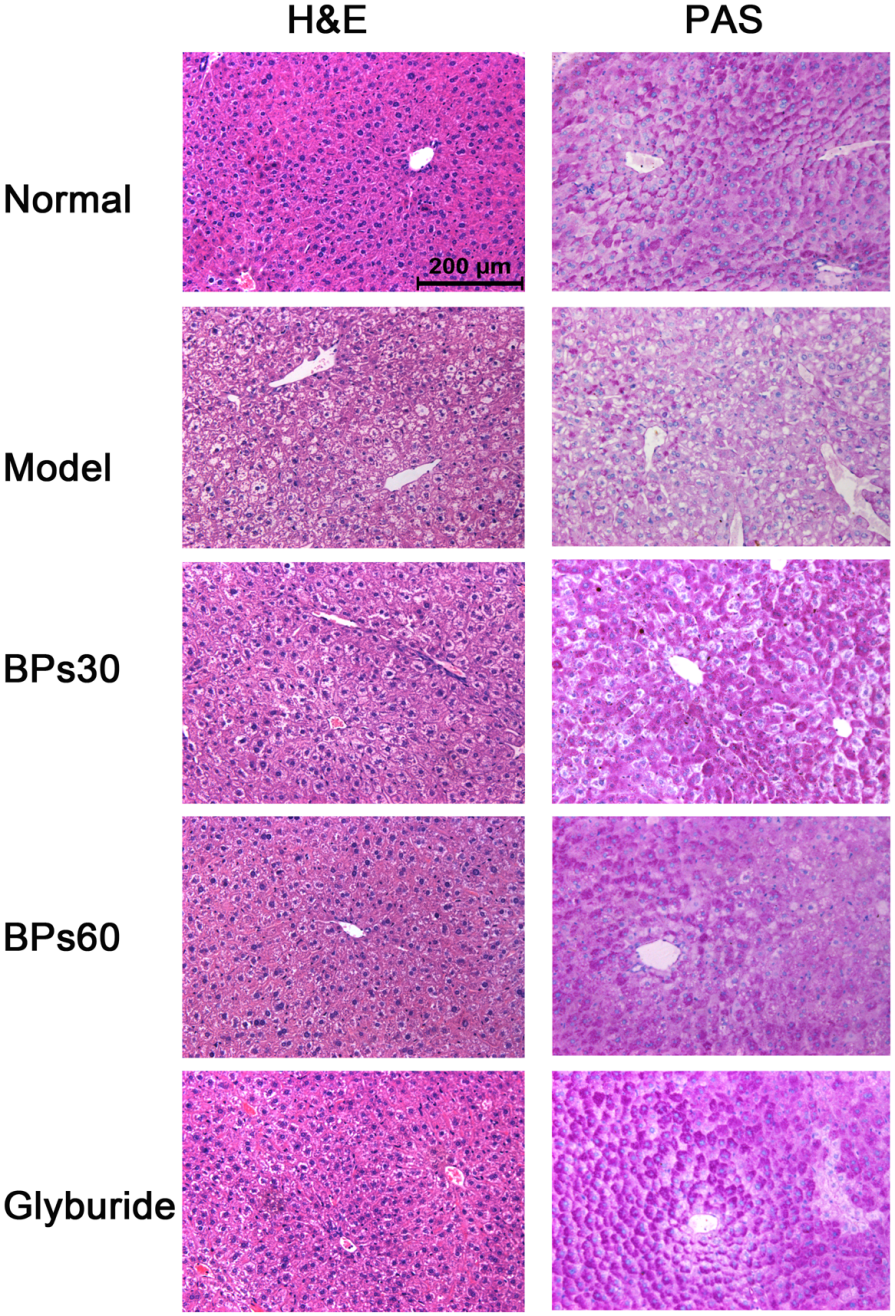
Effect of BPs on histopathology of liver in diabetic mice. Light microscopy 200×. Diabetic mice were treated with BPs (30 and 60 mg/kg/day), glyburide (7.2 mg/kg/day), or vehicle for 35 days.

Pathological changes of the liver and expression of glycogen were observed using H&E and PAS staining methods, respectively. Hepatocytes in the normal mice showed common hepatocyte morphology with abundant glycogen granules in the cytoplasm, distinct cell boundary and a round central nucleus (Fig 6). STZ administration led to severe pathological changes, such as a remarkable glycogen loss, cell vacuolization and focal necrosis. The BPs and glyburide treatments markedly alleviated these histopathological changes. The hepatic architecture of BPs and glyburide group was similar to that in normal liver.

### 2.7 Effect of BPs on oxidative stress in pancreas and liver of diabetic mice

**Fig 7:**
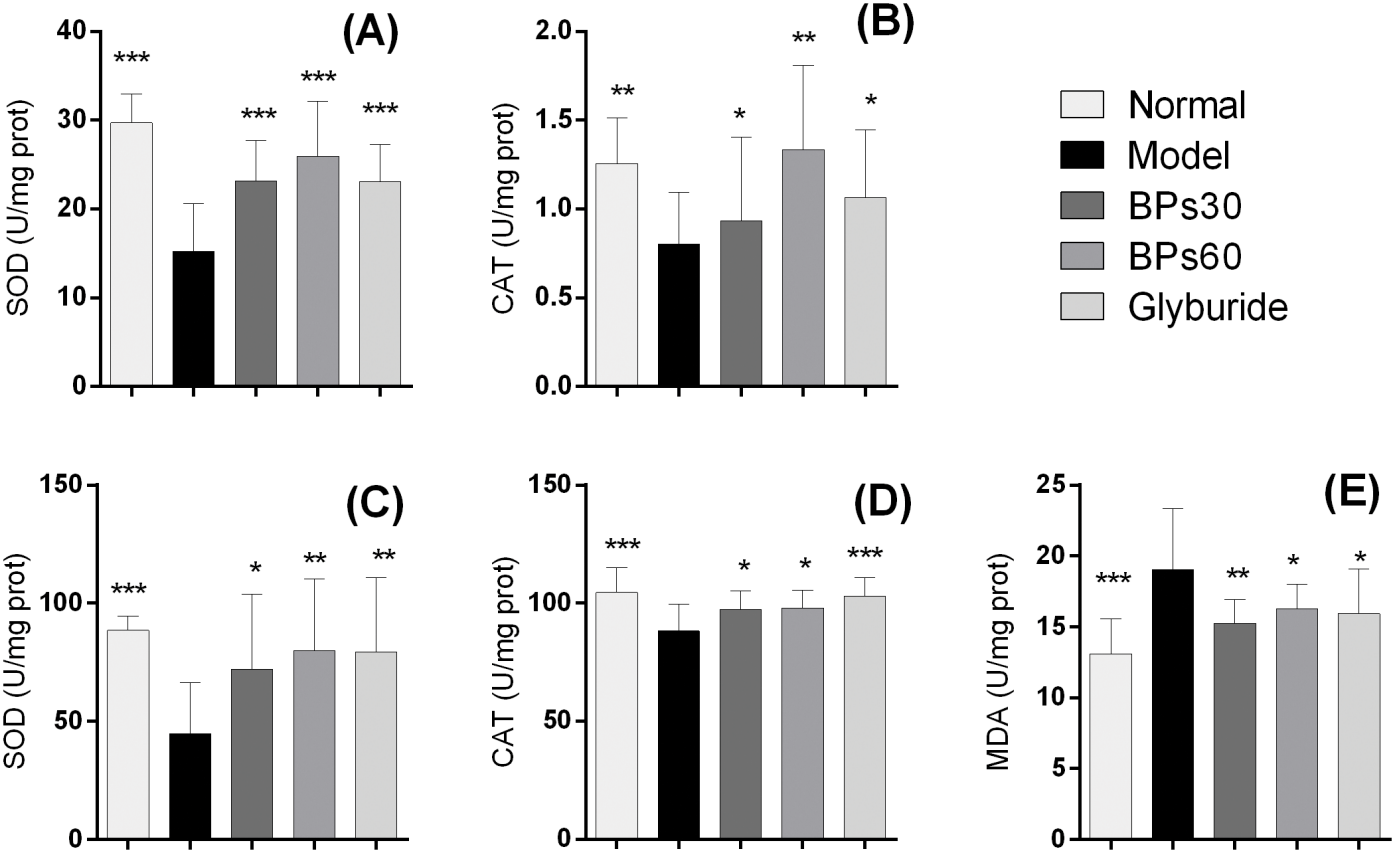
Effect of BPs on oxidative stress in pancreas and liver of diabetic mice. Diabetic mice were treated with BPs (30 and 60 mg/kg/day), glyburide (7.2 mg/kg/day), or vehicle for 35 days. SOD and CAT activity was determined in the pancreas (A, B) and liver (C, D) homogenate, while MDA level (E) was detected in the liver. Data were expressed as means ± S.D.; *P<0.05, **P<0.01, ***P<0.001 compared with model group, analyzed by ANOVA and Fisher’s PLSD.

Significant decreases in the enzymatic activity of SOD (Fig 7A) and CAT (Fig 7B) were observed in the pancreas of vehicle-treated diabetic mice, compared with those in the normal mice (*P*<0.001). Treatments with BPs and glyburide for 35 days resulted in a marked increases in the enzymatic activity (*P*<0.05).

A significant increase in MDA level was observed in the liver of diabetic model mice, compared with that in the normal mice (*P*<0.001). Treatments with BPs and glyburide for 35 days resulted in a marked reduction in MDA in the liver (*P*<0.05) (Fig 7C). Enzymatic activity of SOD (Fig 7D) and CAT (Fig 7E) was significantly reduced in the liver of vehicle-treated diabetic mice, compared with that in the normal mice (*P*<0.001), but significantly elevated by BPs (*P*<0.05) and glyburide (*P*<0.01).

### 2.8 Effect of BPs on inflammatory markers in pancreas and liver of diabetic mice

**Fig 8:**
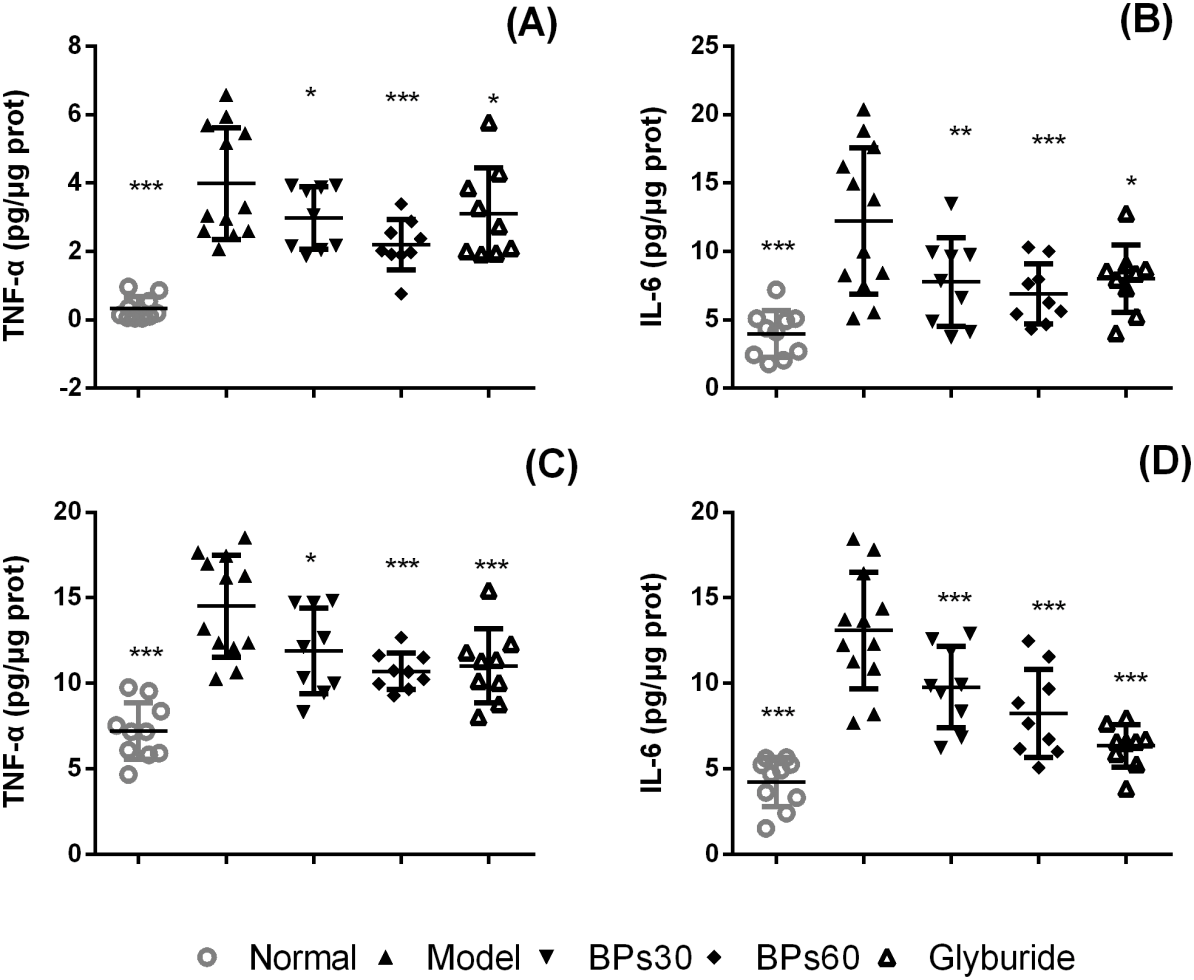
Effect of BPs on inflammatory markers in pancreas and liver of diabetic mice. Diabetic mice were treated with BPs (30 and 60 mg/kg/day), glyburide (7.2 mg/kg/day), or vehicle for 35 days. TNF-α and IL-6 in pancreas (A, B) and liver (C, D) tissues homogenate were determined by ELISA. Data were expressed as means ± S.D.; *P<0.05, **P<0.01, ***P<0.001 compared with model group, analyzed by ANOVA and Fisher’s PLSD.

The levels of TNF-α (Fig 8A) and IL-6 (Fig 8B) in pancreas tissues were significantly elevated in the diabetic mice group compared with the normal mice (*P*<0.001). Treatments with BPs or glyburide significantly reduced TNF-α and IL-6 production in comparison with the vehicle-treated diabetic mice (*P*<0.05).

The levels of TNF-α (Fig 8C) and IL-6 (Fig 8D) in liver tissues were also significantly elevated in the diabetic mice group compared with the normal mice (*P*<0.001). Treatments with BPs or glyburide significantly reduced TNF-α and IL-6 production in comparison with the vehicle-treated diabetic mice (*P*<0.05).

## Discussion

In recent decades, prevention and treatment of diabetic complications have been considered the most important practices for the general care of diabetic patients [8, 27]. Diabetes mellitus and its complications are associated with oxidative stress and chronic low-grade inflammation [2, 3]. Thus, the anti-oxidative and anti-inflammatory activities of traditional antidiabetic drugs may contribute to antidiabetic effects [4, 5, 28]. However, synthetic hypoglycemic drugs, including insulin biguanides, sulphonylureas and α-glucosidase inhibitors, although can control blood glucose levels, have moderate therapeutic effects with many severe side effects [29]. Therefore, searching for drugs with antidiabetic activity and fewer side effects has been a focus in the efforts to manage the disease and its complications.

Polysaccharides have attracted much attention for antidiabetic therapy, owing to their therapeutic properties and relatively low toxicity [30, 31]. It has been suggested that herbal polysaccharides lower blood glucose via multiple mechanisms: 1) increasing content of liver glycogen, 2) stimulating release of insulin, 3) influencing activities of digestive enzymes, and 4) attenuating oxidative stress [32]. In this study, BPs showed some antidiabetic properties similar with other herbal polysaccharides, such as increasing liver glycogen, improving serum insulin and attenuating oxidative stress. Moreover, BPs inhibited inflammation in pancreas and liver which may also contribute to its antidiabetic effects.

STZ is an antibiotic produced by *Streptomyces achromogenes*, and is widely used to induce diabetic models in rodents [28, 33]. In this study, the STZ-induced diabetic mice exhibited typical characteristics, including polydipsia, polyphagia, high blood glucose level, moderate lipid abnormalities and partial islets reservation, which were similar to the characteristics in type 2 diabetes in humans. In order to validate the mouse model of diabetes, we chose glyburide as the positive drug. Glyburide is widely used in type 2 diabetes through its action on stimulating insulin release from the β-cells [34]. The results in this study also supported this conclusion. Our results also showed that glyburide exerted modulatory effects on the glucose and lipid metabolism, and protection of the pancreas and liver from exogenous insults [33, 35].

Weight loss is a major characteristic of diabetes mellitus. It may be a result of protein wasting because of lack of carbohydrates for energy [36]. In this study, BPs maintained the body weight of diabetic mice during treatment, suggesting that BPs may normalize energy metabolism in tissues.

Hyperlipidemia (high serum cholesterol and triglycerides) is associated with diabetes [35]. The present study showed increased serum lipid concentrations in STZ-induced diabetic mice. Treatments with BPs improved the levels of serum LDL-C and HDL-C, but exerted no effects on serum triglycerides and total cholesterol. These results suggested that BPs only moderately influenced lipid metabolism, so the therapeutic effect of BPs on diabetic mice might not mainly be attributed to the regulation of lipid abnormalities.

In diabetic individuals, hepatic glucose production is elevated, thus, the rate of glucose appearance exceeds the rate of glucose disappearance in the circulation, causing postprandial hyperglycemia. To control the level of blood glucose, insulin promotes peripheral glucose utilization, inhibits glucagon secretion, enhances glycogenesis and inhibits glycogenolysis [37]. However, diabetes mellitus impairs the normal function of islets to secrete sufficient insulin [38]. In this study, the diabetic mice showed lower serum insulin, and higher blood and urine glucose levels. BPs treatment significantly regulated serum insulin levels, and controlled glucose levels in diabetic mice. The capability of BPs to decrease postprandial blood glucose levels may relate to improving the secretion of insulin from residual islet β-cells.

Streptozotocin causes the dysfunction of islet β-cells, including induction of pancreatic cell apoptosis and inhibition of insulin gene expression and insulin synthesis [39]. Apoptosis of pancreatic β-cells is commonly regarded as the primary cause of hyperglycemia [40]. Caspase are specifically activated in cells for apoptosis, and caspase 8 and caspase 3 are regarded as the key executioners during apoptosis. Our present study revealed that BPs treatment markedly suppressed caspase 8 and caspase 3 activation in pancreas of diabetic mice. In addition, the diabetic mice showed shrunken islets, fewer insulin expression and severe apoptosis in islets cells due to streptozotocin injury. BPs exerted the protection of existing islet β-cells function and prevention of β-cells from apoptosis. Therefore, we suggested that BPs reduced postprandial serum glucose levels due to the protection of pancreas in diabetic mice.

The liver is sensitive to insulin in modulating glucose metabolism, and thus, can be severely affected by diabetes. In this study, significant reductions in the glycogen content and severe pathologic damages were observed in the livers of the diabetic mice. BPs normalized the liver pathology and its function to synthesize glycogen. The question of whether the effects BPs on liver are due to direct action on hepatocytes or indirect effect on insulin secretion remains to be addressed.

Hyperglycemia induces oxidative stress, which plays a vital role in the pathogenesis of diabetes and diabetic complications in humans, as well as STZ-induced diabetic mice [41–43]. Some polysaccharides exhibited antidiabetic properties by suppressing cell oxidative stress, including inhibiting the production of MDA and improving the activity of antioxidant enzymes [42]. Our present study showed that anti-oxidative activity of BPs included improvement of the enzymatic activity of SOD and CAT both in the pancreas and liver, as well as reductions of the MDA level in liver. These results suggest that BPs also act as antioxidants to protect cells from oxidative damages.

In diabetes, oxidative stress increases the excretion of inflammation-related cytokines, such as IL-6 and TNF-α [44, 45]. TNF-α inhibits the function of mitochondrial electron respiratory chain and causes excess generation of reactive oxygen species (ROS). This elevated reactive oxygen species, in a feedback fashion, further activates inflammatory signaling cascades and exacerbates local inflammation and tissue injury [45]. IL-6 can activate the NF-κB signaling pathways in hepatocytes and subsequently exacerbate local inflammation [44]. In the present experiment, the levels of IL-6 and TNF-α in the pancreas and liver tissues of diabetic mice were significantly increased, while BPs significantly decreased the pro-inflammatory cytokines levels. These observations suggested that BPs suppressed the expression of pro-inflammatory cytokines and production of ROS, thus, blocked the malignant ROS-inflammatory cycle both in the pancreas and liver. Therefore, we suggested that the anti-oxidative and anti-inflammatory characteristics of BPs contributed to its pancreas and liver protection and antidiabetic actions.

## Conclusions

Polysaccharides from *Bupleurum smithii* var. *parvifolium* ameliorated diabetes, protected pancreatic β cells and liver hepatocytes from oxidative and inflammatory insults. BPs is therapeutic candidate for diabetes and diabetic complications.
